# An Altered Immune Response, but Not Individual Cationic Antimicrobial Peptides, Is Associated with the Oral Attenuation of Ara4N-Deficient *Salmonella enterica* Serovar Typhimurium in Mice

**DOI:** 10.1371/journal.pone.0049588

**Published:** 2012-11-15

**Authors:** Kristi L. Strandberg, Susan M. Richards, Rita Tamayo, Linh T. Reeves, John S. Gunn

**Affiliations:** 1 Center for Microbial Interface Biology, The Ohio State University, Columbus, Ohio, United States of America; 2 Department of Microbiology and Immunology, University of Texas Health Science Center at San Antonio, San Antonio, Texas, United States of America; Indian Institute of Science, India

## Abstract

*Salmonella enterica* serovar Typhimurium (*S.* Typhimurium) uses two-component regulatory systems (TCRS) to respond to stimuli in the local microenvironment. Upon infection, the *Salmonella* TCRSs PhoP-PhoQ (PhoPQ) and PmrA-PmrB (PmrAB) are activated by environmental signals in the intestinal lumen and within host cells. TCRS-mediated gene expression results in lipopolysaccharide (LPS) modification and cationic antimicrobial peptide resistance. The PmrA-regulated *pmrHFIJKLM* operon mediates 4-amino-4-deoxy-L-arabinose (Ara4N) production and attachment to the lipid A of LPS. A Δ*pmrF S*. Typhimurium strain cannot produce Ara4N, exhibits increased sensitivity to cationic antimicrobial peptide (CAMP)-mediated killing, and attenuated virulence in mice upon oral infection. CAMPs are predicted to play a role in elimination of *Salmonella*, and may activate PhoPQ and PmrAB in vivo, which could increase bacterial resistance to host defenses. Competition experiments between wild type (WT) and Δ*pmrF* mutant strains of *S*. Typhimurium indicated that selection against this mutant first occurs within the intestinal lumen early during infection. However, CRAMP and active cryptdins alone are not responsible for elimination of Ara4N-deficient bacteria in vivo. Investigation into the early immune response to Δ*pmrF* showed that it differed slightly from the early immune response to WT *S.* Typhimurium. Further investigation into the early immune response to infection of Peyer’s patches suggests a role for IL-13 in the attenution of the *ΔpmrF* mutant strain. Thus, prominent CAMPs present in the mouse intestine are not responsible for the selection against the Δ*pmrF* strain in this location, but limited alterations in innate immune induction were observed that affect bacterial survival and virulence.

## Introduction

Upon infection, bacteria must evade a powerful arsenal of host immune cells and antimicrobial factors to survive in vivo. Cationic antimicrobial peptides (CAMPs) are diverse, amphipathic, innate defense molecules produced by epithelial cells and phagocytes of many organisms that permeabilize negatively charged bacterial membranes to cause cell leakage and death [1], [2], [3]. In addition to playing a direct role in bacterial killing, several CAMPs have immunomodulatory properties and help regulate other host immune responses that participate in elimination of invading pathogens [2], [3], [4].

*Salmonella enterica* use two-component regulatory systems (TCRSs), including PhoP-PhoQ (PhoPQ) and PmrA-PmrB (PmrAB), to modify gene expression in response to the local microenvironment [5], [6], [7]. *Salmonella enterica* serovar Typhimurium (*S*. Typhimurium) sensing of environmental signals through the inner membrane-bound sensor kinase PhoQ results in activation of the PhoPQ regulon, including genes whose products mediate lipopolysaccharide (LPS) modification [8], [9]. It has been demonstrated that CAMPs can serve as activation signals for *Salmonella* PhoQ in vitro and in vivo [8], [9], [10]. *Salmonella* TCRS-mediated detection of specific environmental factors, which may include CAMPs, during infection likely allows bacteria to mediate rapid changes in gene expression that promote survival in the presence of these and other host immune defenses.

PmrA-regulated lipid A modifications promote CAMP resistance by reducing the anionic charge of the bacterial outer membrane. Activation of the *S.* Typhimurium PmrA-regulated *pmrE* gene and the *pmrHFIJKLM* operon leads to aminoarabinose (Ara4N) production and its attachment to lipid A [11], [12]. As previously reported and confirmed by this work, *S*. Typhimurium Δ*pmrA* and Δ*pmrF* strains cannot produce Ara4N, are more sensitive to CAMP killing than are wild type (WT) bacteria and have attenuated virulence in mice upon oral, but not intraperitoneal (IP), infection [11], [12]. These Ara4N-deficient mutants also exhibit a survival defect compared to WT *Salmonella* in competition assays in vivo [11], [12]. The specific host factors responsible for the decreased pathogenesis of these mutants remain unknown.

The objective of this work was to investigate the basis of the oral virulence defect of Ara4N-deficient *S.* Typhimurium, as well as the host gut immune responses that must be overcome by *S.* Typhimurium during the course of infection. The experiments described here were designed to identify the in vivo signals that mediate the killing of orally-delivered CAMP-sensitive mutants, and to investigate differences in early immune responses to WT and CAMP-senstive mutants. We hypothesize that CAMPs and the differential innate immune signals generated by the Ara4N-deficient mutant may be involved in elimination of this strain during oral infection.

Our results suggest that neither of the tested CAMPs individually play a direct role in killing of LPS modification mutants in vivo. However, other immune responses appear to play a pivitol role in the attenuation of Ara4N-deficient *S.* Typhimurium. The findings described here promote a better understanding of the genetic basis of *Salmonella* pathogenesis, the role of LPS modifications in resistance to CAMPs and the arsenal of host innate immune responses induced upon bacterial infection.

## Materials and Methods

### Bacterial Strains and Growth Conditions

*S.* Typhimurium strains used in this study are listed in Table 1. Luria-Bertani (LB) broth and agar were used for strain maintenance. When appropriate, antibiotics were added at the following concentrations: 25 µg/ml chloramphenicol, 45 µg/ml kanamycin, 1 mg/ml streptomycin, or 15 µg/ml tetracycline. *Salmonella* recovered from mouse tissues were plated on either LB agar containing appropriate antibiotics or *Salmonella-Shigella* (SS) agar. *S.* Typhimurium was grown in a rotating drum at 37°C with aeration to late log phase (OD_600_ = 0.9) for virulence assays and competition assays. Prior to infection, the strains were equalized by O.D. in 1X phosphate buffered saline (PBS).

**Table 1:** *Salmonella enterica* serovar Typhimurium strains.

Strain	Relevant characteristics	Source or reference
JSG210	ATCC14028s (CDC)	ATCC
JSG224	ATCC14028s; phoN2 ZXX::6251dTn10-CAM (85% linked to phoN) = CS019 (Cam)	[7]
JSG421	ATCC14028s; pmRA::Tn10d (Tet)	[5]
JSG435	ATCC14028s; PrmA^c^ *pmrA*505 zjd::Tn10d-CAM (Cam)	[5]
JSG542	CS019+ Strep resistance cassette (Strep/Cam)	This study
JSG790	ATCC14028s; *prgH*::Tn*phoA* (Kan/Cam)	[33]
JSG1049	ATCC14028s; *pmrF*::Tn10d (Tet)	[11]
JSG1525	ATCC14028s; PmrA-reg *yibD*::MudJ (phage from JSG897 transduced into JSG210) (Kan)	[34]

### *S*. Typhimurium Virulence Assays

In vivo experiments were performed with 6–10 week old female BALB/c mice from Harlan Sprague Dawley (Indianapolis, IN). Mice were housed and used in strict accordance with guidelines established by The Ohio State University Institutional Animal Care and Use Committee (IACUC), and all efforts were made to minimize animal suffering. Food and water were removed from the cages of mice intended for oral infection 4 hrs prior to infection to reduce the intestinal contents and promote *Salmonella* invasion. Mice were infected via the oral or IP route with WT (JSG210), PmrA^c^ (JSG435), or Δ*pmrF* (JSG1049) *S.* Typhimurium (3–5 mice per condition). *S.* Typhimurium strains were equalized as described above and diluted in cold PBS and then mice were infected via the oral route with 1×10^6^ CFU, 5×10^5^ CFU or 1×10^4^ CFU or via the IP route with 1×10^2^ CFU (100 µL of the appropriate equalized strain listed above) of WT, Δ*pmrF,* or PmrA^c^ *S.* Typhimurium. IL-13 KO BALB/c mice originated from the McKenzie laboratory [13] were obtained as a gift from the Rothenberg Laboratory at Cincinnati Children’s Hosptial. IL-13 KO mice were housed and bred in accordance with guidelines established by IACUC. Food and water were removed from cages prior to infection, as described above. IL-13 KO mice were infected by the oral route with 100 µl PBS containing 10^4^ CFU WT or Δ*pmrF S.* Typhimurium. After infection, food and water were returned to all cages and mice were observed daily to monitor survival and signs of morbidity.

### Competition Assays in CAMP-deficient Mice

In vivo competition experiments were performed with 6–10 week old female BALB/c mice from Harlan Sprague Dawley and CRAMP knockout (KO) mice initially bred in and obtained from the laboratory of Dr. Bradford McGwire at The Ohio State University. C57BL/6 and B6.129-Mmp7 (MMP7 KO; Matrilysin-deficient) mice (Jackson Laboratories, Bar Harbor, Maine) were infected via the oral route with a 1∶1 mixture of WT/WT or Δ*pmrF*/WT *S.* Typhimurium (3–5 mice per condition). Mice were infected via the oral route with 100 µL of a 1∶1 mix containing approximately 5×10^5^ CFU/50 µL or 1×10^8^ CFU/50 µL of WT (JSG210 or JSG224) *S*. Typhimurium and either Δ*pmrF* or another *S.* Typhimurium strain that does not exhibit a virulence defect (JSG1525 or JSG542) as a positive control (3–5 mice per condition). Mice were monitored daily and sacrificed at 12, 48 and 96 hours p.i. for recovery of bacteria from infected organs. To distinguish between competing strains recovered from the mouse organs, they were marked with different antibiotic resistances. The Δ*pmrF S.* Typhimurium strain contained a tetracycline resistance cassette.

For the initial competition assays, FvB (WT) and background-matched CRAMP KO mice were infected with a 1∶1 mixture of WT/WT or Δ*pmrF*/WT *S*. Typhimurium. The cecum and one inch of the distal ileum were removed from each mouse at 12 and 48 hours p.i. for recovery of *S.* Typhimurium. Subsequent competition assays were performed in BALB/c (WT) and background-matched CRAMP KO mice. In these experiments, the luminal contents, Peyer’s patches, mesenteric lymph nodes (MLN) and spleen were removed from each mouse (3 mice per strain at each time point) at 48 and 96 hours p.i. for recovery of *S*. Typhimurium. Experiments in both FVB and BALB/c mice were performed at least three times each.

C57BL/6 (WT) and background-matched MMP7 KO mice were infected via the oral route with a 1∶1 mixture of WT and WT (JSG210 or JSG224) or Δ*pmrF* (JSG1049) *S*. Typhimurium. For these experiments, competing bacteria strains were recovered from the liver, spleen, luminal contents, Peyer’s patches, or mesenteric lymph nodes (MLN) at 48 and 96 hours p.i. Tissue samples were homogenized in 1 mL of cold 1X PBS, diluted and plated on LB, *Salmonella*-*Shigella* (SS) Agar and/or appropriate antibiotic-containing media (based on the antibiotic resistance of the competing strains) to detect survival of competing strains in each organ over time in mice with or without CRAMP or mature cryptdins. Total CFU/mL of each bacteria strain recovered from each mouse tissue/organ was calculated for determination and comparison of the Competitive Index (CI) ratio of mutant:WT or WT:WT *S*. Typhimurium at each time point. Alternatively, for some experiments, homogenized and diluted tissues samples were plated on LB and incubated overnight at 37°C. Resulting colonies were patched on LB and appropriate antibiotic-containing media. Patch plates were incubated overnight at 37°C and antibiotic-sensitive and resistant colonies were counted the following day to determine the percentage of WT and Δ*pmrF S*. Typhimurium recovered from mice. Statistical significance was determined by student’s t-test comparing CI ratios of WT and Δ*pmrF S*. Typhimurium recovered from WT or either CRAMP KO or matrilysin-deficient mice.

### Early Timepoint Mouse Infection Studies

BALB/c mice were infected orally with 0.1 ml PBS containing 10^8^ CFU of WT (JSG210), Δ*pmrA,* or Δ*pmrF S.* Typhimurium. As a negative control, mice were mock infected with 0.1 ml PBS. Mice were euthanized at 12, 24, and 48 hours post infection, and three Peyer’s patches were collected from each mouse for RNA isolation or histological studies. Peyer’s patches collected for RNA isolation were immediately placed in 1 ml TRIzol (Invitrogen, Carlsbad, CA). Peyer’s patches collected for histological studies were placed in 10% formalin (Fisher, Waltham, MA).

### qRT-PCR Detection of Immune-related Gene Expression

Changes in mouse immune-related gene expression during infection with *S.* Typhimurium were detected using a TaqMan® Mouse Immune Array with the 7900HT Fast Real-Time PCR System (Applied Biosystems, Carlsbad, CA). For these studies, RNA was isolated from murine Peyer’s patches after 12, 24, or 48 hrs of infection with *S.* Typhimurium (or mock infection with PBS). Each experimental sample represents three pooled Peyer’s patches collected from one mouse. Prior to RNA isolation, the 3 pooled Peyer’s patches were homogenized in 1 ml TRIzol. RNA was isolated from Peyer’s patches using the RiboPure Kit (Applied Biosystems). Fold change was calculated using the Pfaffl method (2^−ΔΔCt^) with infected Peyer’s patches being compared with PBS mock-infected Peyer’s patches [14].

### Histology of Mouse Peyer’s Patches

Peyer’s patches were collected from orally-infected BALB/c mice after 12, 24 and 48 hrs of infection with 10^8^ CFU of WT, Δ*pmrA,* or Δ*pmrF S.* Typhimurium. Peyer’s patches were also collected from PBS mock-infected BALB/c mice after 12, 24, and 48 hrs. Peyer’s patches were collected from IL-13 KO BALB/c mice infected with 10^8^ CFU of WT or Δ*pmrF S.* Typhimurium, or PBS mock-infected mice. Peyer’s patches were fixed in 10% formalin, and were processed, sectioned, and stained by the Comparative Pathology & Mouse Phenotyping Shared Resource at The Ohio State University. Hematoxylin & Eosin (H&E) staining was performed to allow for visualization of general tissue architecture. Immunohistochemistry was performed to stain for infiltrating neutrophils (Ly6G) and macrophages (F4/80).

## Results

### Aminoarabinose-deficient *S*. Typhimurium Mutants Exhibit a Virulence Defect in Susceptible Mice

**Figure 1:**
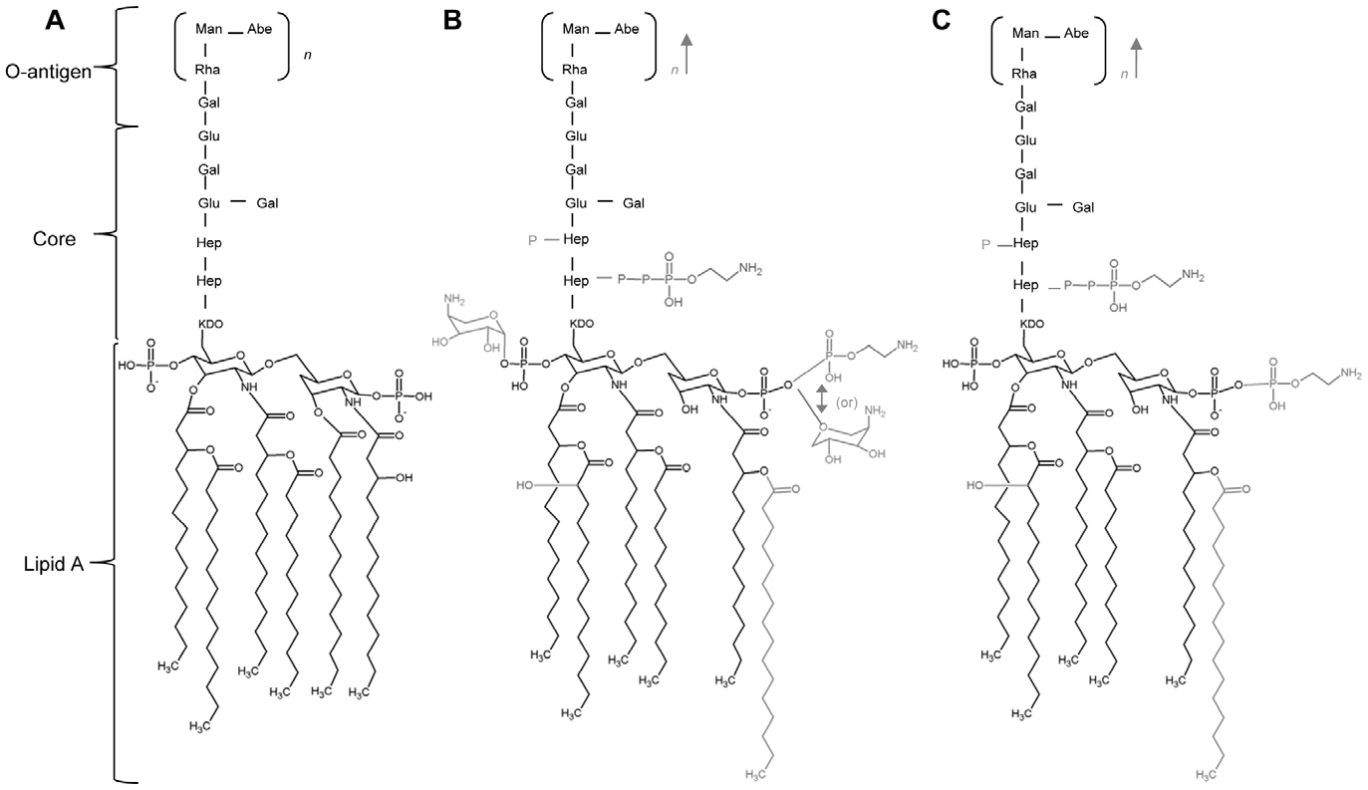
*S*. Typhimurium PhoP-PhoQ and PmrA-PmrB modified LPS. (A.) Unmodified LPS of *S.* typhimurium. Unmodified LPS predominates when *S.* Typhimurium is grown in vitro in LB broth or on plates. (B.) PhoPQ- and PmrAB-modified *S.* Typhimurium LPS. This highly modified form of LPS is found when *S.* Typhimurium in grown in vivo. (C.) LPS modifications capable of being made by the Ara4N-deficient strain of *S.* Typhimurium.

WT *S*. Typhimurium can modify the LPS with Ara4N upon activation of PhoQ and/or PmrB (Figure 1B). While grown in rich media with high Mg^2+^ concentrations, the unmodified form of *S.* Typhimurium LPS predominates (Figure 1A), however, when in vivo, *S.* Typhimurium LPS is highly modified (Figure 1B). Ara4N-deficient *Salmonella* has been shown to have a virulence defect in mice upon oral infection [12]. A Δ*pmrF S.* Typhimurium strain (JSG1049) specifically lacks the Ara4N modification due to a polar *pmrF*::Tn*10d* insertion, which prevents expression of the majority of the *pmrH* operon and subsequent Ara4N production and lipid A modification (Figure 1C). A PmrA^c^ strain of *S*. Typhimurium (JSG435) has high levels of Ara4N and other PmrA-regulated LPS modifications due to a point mutation at *pmrA*505 that causes unregulated constitutive activation of PmrA [15].

**Figure 2:**
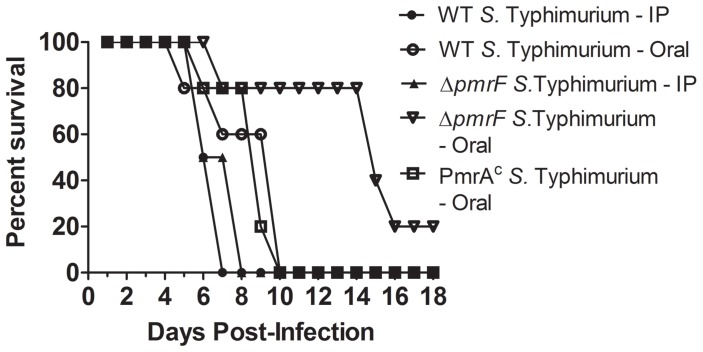
Survival analysis of BALB/c mice infected with WT or mutant *S*. Typhimurium. BALB/c (WT) mice were infected via the oral route with either 10^4^ CFU of WT, Δ*pmrF* or PmrA^c^ *S*. Typhimurium or with 10^2^ CFU of WT or Δ*pmrF S*. Typhimurium via the IP route. The graph depicts representative results from the virulence assays, which were performed in triplicate.

Strains were chosen to compare the virulence of *Salmonella* that produce different levels of Ara4N-modified lipid A. Experiments were performed with 5×10^5^ CFU (data not shown) or 1×10^4^ CFU of *S*. Typhimurium delivered via the oral route [16], as well as IP infection with 1×10^2^ CFU of WT or Δ*pmrF S*. Typhimurium. Mice that were infected via the IP route with WT or Δ*pmrF S*. Typhimurium survived for 7–8 days and mice infected with 1×10^4^ CFU of WT or PmrA^c^ *S*. Typhimurium via the oral route survived for 6–10 days (Figure 2). The majority of the mice orally infected with Δ*pmrF S*. Typhimurium survived for 7–16 days, but one mouse remained healthy after 18 days, which served served as the endpoint for the experiment. Therefore, the Δ*pmrF* strain is less virulent by the oral route of infection than both WT *S*. Typhimurium and the PmrA^c^ mutant and all three strains are equally virulent by the ip route (Figure 2) [12]. The results confirmed and extended the finding that Ara4N-deficient *S*. Typhimurium exhibit a survival defect compared to WT bacteria upon oral, but not IP infection [12].

### CRAMP does Not Play a Significant Role in Selection Against *S*. Typhimurium in vivo

Δ*pmrF S*. Typhimurium demonstrate WT virulence upon IP infection, which allows bacteria to bypass the stomach and small intestine and be taken up directly by host cells in the peritoneal cavity. Therefore, environmental factors that could be responsible for the reduced virulence of Δ*pmrF S*. Typhimurium upon oral infection were hypothesized to be located along the route of infection before the bacteria enter macrophages.

The Δ*pmrF S*. Typhimurium was expected to exhibit a virulence defect somewhere during the early stages (relating to time and location) of host infection. Previous research examined the harsh environment of the stomach, specifically the acidic conditions, for a role in selection against mutant bacteria. However, WT and mutant bacteria survived equally well upon transit through the pH-buffered mouse stomach and Δ*pmrF S*. Typhimurium did not show a growth defect at a range of different pH conditions in vitro (data not shown). Because an invasion deficiency could also affect bacterial dissemination, Ara4N-deficient *S*. Typhimurium was examined for invasion of immortalized human intestinal epithelial cells (T84 cells) in vitro. While the rate of invasion was slightly lower than that of WT *S.* Typhimurium, similar levels of invasion were observed (data not shown). Bile sensitivity was also examined as the *Escherichia coli* PmrA regulon has been implicated in bile resistance [17]. We found no difference in susceptibilities between the WT and Ara4N-deficient S. Typhimurium (data not shown).

**Figure 3:**
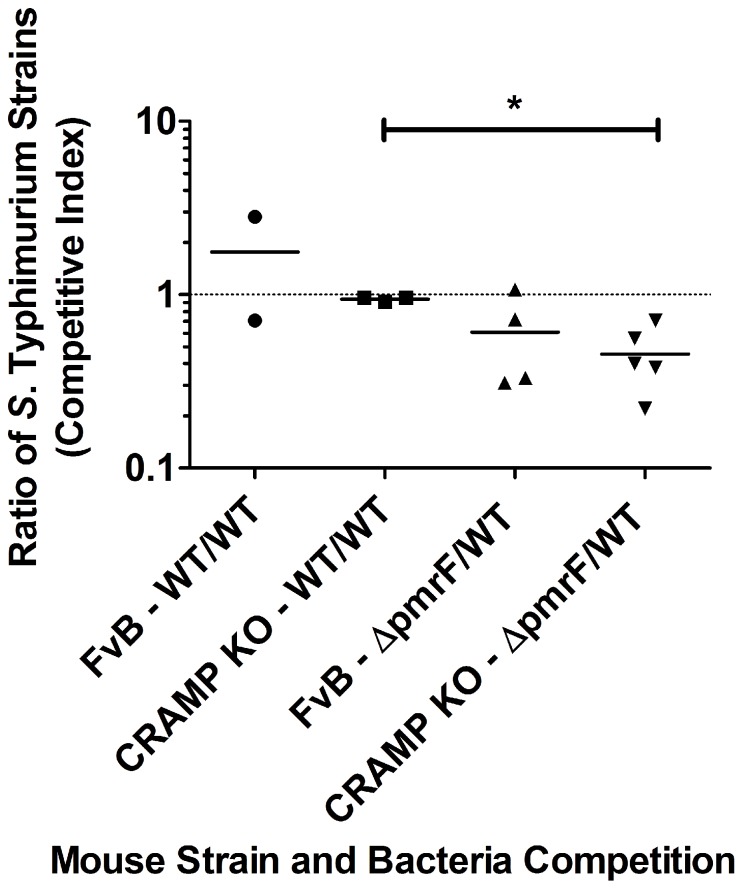
Survival of WT and Δ*pmrF S*. Typhimurium in WT and CRAMP KO FvB mice. FvB (WT) and background-matched CRAMP KO mice were infected via the oral route with a 1∶1 mixture of WT and Δ*pmrF S*. Typhimurium (or a 1∶1 mixture of two strains with WT virulence) containing 5×10^5^ CFU of each strain. Graphed values represent the competitive index (CI) ratio (output ratio/input ratio) of Δ*pmrF*/WT (or WT/WT) bacteria recovered from the caecum and ileum of each mouse after 48 hrs of infection. Each value graphed as the same shape represents the CI ratio calculated from the specified organ of one WT or CRAMP KO mouse; (*) indicates p≤0.005.

To determine if the antimicrobial peptide CRAMP is an in vivo factor involved in elimination of Δ*pmrF S*. Typhimurium, preliminary competition experiments were performed in susceptible FvB mice, or background matched CRAMP KO mice that were infected via the oral route with a 1∶1 mixture of WT and Ara4N-deficient *S*. Typhimurium for 12 or 48 hrs. Few bacteria were recovered after 12 hrs of infection (data not shown). As expected, the control WT/WT competition experiments centered around 1 indicating equal competition in both FvB and CRAMP KO mice (Figure 3). Δ*pmrF S.* Typhimurium exhibited an obvious survival defect as expected in both FvB and CRAMP KO mice after 48 hrs p.i. (reaching significance [p value of 0.005] in the CRAMP KO background only). However, there was no significant difference in the Δ*pmrF*/WT CI observed in FvB mice compared to CRAMP KO mice, suggesting that CRAMP was not responsible for the competetive disadvantage of the Δ*pmrF* strain.

**Figure 4:**
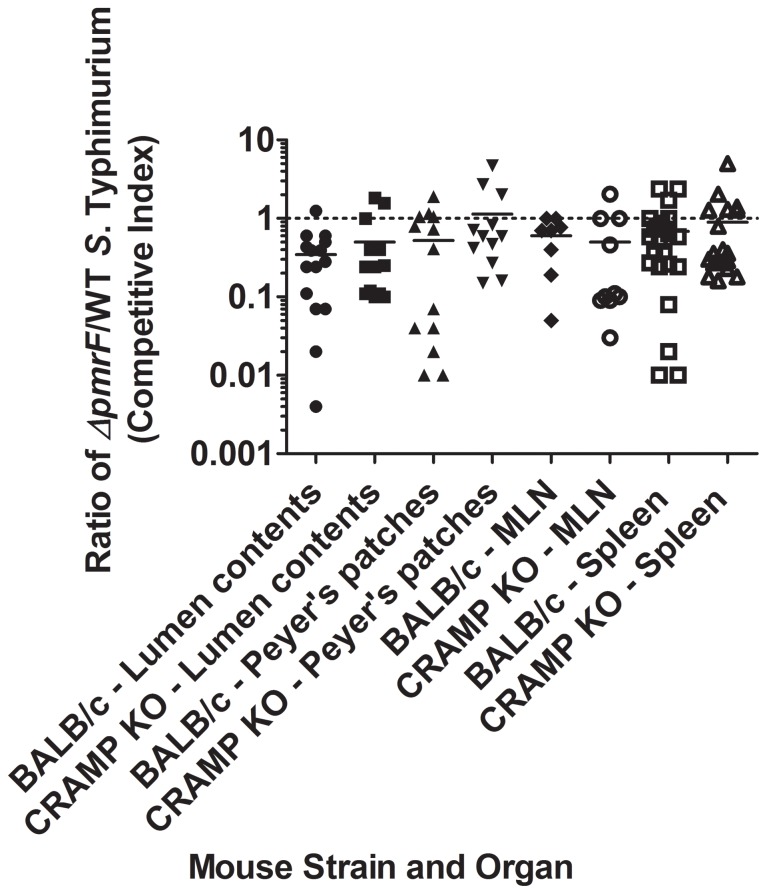
Survival of WT and Δ*pmrF S.* Typhimurium in BALB/c and CRAMP KO mice. BALB/c (WT) and background-matched CRAMP KO mice were infected via the oral route with a 1∶1 mixture of WT *S*. Typhimurium and Δ*pmrF* bacteria containing 10^8^ CFU of each strain. Bacteria were recovered from the lumen contents, Peyer’s patches, mesenteric lymph nodes (MLN) and spleen from 24 BALB/c and 22 CRAMP KO mice 96 hrs. p.i. Graphed values represent the competitive index (CI) ratio (output ratio/input ratio) of Δ*pmrF/*WT bacteria recovered from each mouse organ (*Salmonella* was not recovered from each organ). Each value graphed as the same shape represents the CI ratio calculated from the specified organ of one WT or CRAMP KO mouse. There are no significant differences between any of the observed average CI ratios (p≥0.05).

Additional competition experiments were performed using WT BALB/c mice and background-matched CRAMP KO mice infected with 1×10^8^ CFU WT or Ara4N-deficient *S.* Typhimurium for 48 or 96 hrs. Few bacteria were recovered at 48 hrs p.i. (data not shown), but the expected selection against Δ*pmrF S*. Typhimurium (CI generally less than 1) was observed in both WT and CRAMP KO mice at 96 hrs p.i. (Figure 4). Δ*pmrF S*. Typhimurium demonstrated reduced survival beginning in the intestinal lumen (before epithelial cell invasion) and generally were not able to recover to WT levels of survival as the infection progressed to deeper tissues. However, statistical analysis showed no significant difference between the competitive indices observed in the different tissues between BALB/c and CRAMP KO mice, again demonstrating that CRAMP was not responsible for the competetive disadvantage of the Δ*pmrF* strain.

### Intestinal α-defensins do not Play a Significant Role in Selection Against *S*. Typhimurium in vivo

The role of intestinal α-defensins in selection against Δ*pmrF S*. Typhimurium was examined by performing competition assays in C57BL/6 (WT) and background-matched MMP7 KO (Matrilysin-deficient) mice. Matrilysin is a cysteine protease produced in and secreted from small intestinal crypts that cleaves the propeptide of murine α-defensins, called cryptdins, into the active form [18], [19]. MMP7 KO mice still produce the propeptide, but this α-defensin precursor is not reported to be secreted or functional in vivo.

**Figure 5:**
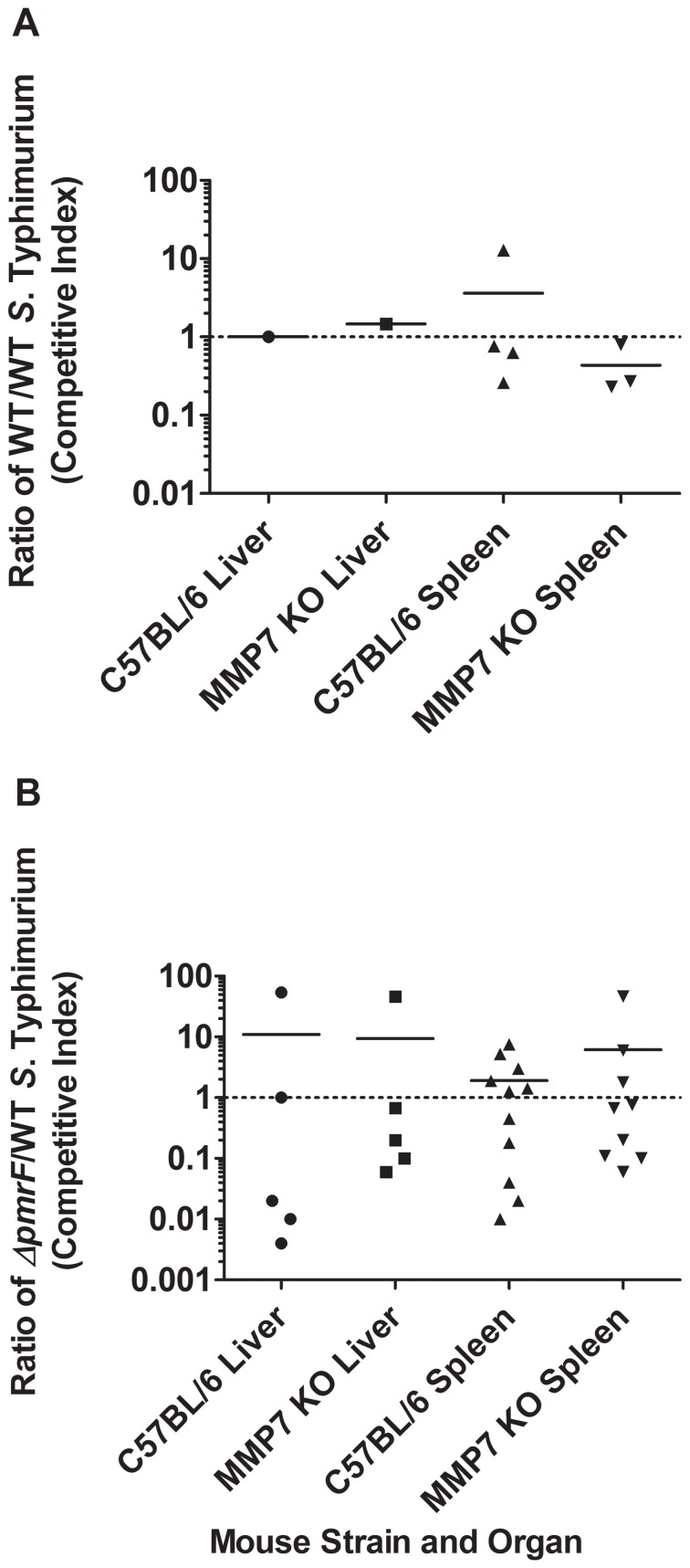
In vivo survival of WT and Δ*pmrF S*. Typhimurium in C57BL/6 and MMP7 KO mice. C57BL/6 (WT) and background-matched MMP7 (Matrilysin-deficient) mice were infected via the oral route with a 1∶1 mixture of WT and WT *S.* Typhimurium, or WT and *ΔpmrF S*. Typhimurium containing 10^8^ CFU of each strain. Graphed values represent the competitive index (CI) ratio output ratio/input ratio) of WT/WT (A.) or Δ*pmrF/*WT (B.) recovered from the livers and spleens of MMP7 KO mice or C57BL/6 mice at 96 hrs. p.i. Each value graphed as the same shape represents the CI ratio calculated from the specified organ of one WT or MMP7 KO mouse. There are no significant differences between any of the observed average CI ratios (p≥0.05).

Competition assays in C57BL/6 vs. MMP7 KO mice were performed to determine if α-defensins play a role in selection against Ara4N-deficient *Salmonella*. These experiments tested survival of Δ*pmrF* compared to WT *S*. Typhimurium. While the means are not generally reflective of this due to some outliers, in the majority of mice, Δ*pmrF* showed a competition defect compared to WT *S*. Typhimurium in both the liver and spleen consistant with our previous results. This was true regardless of the presence or absence of functional α-defensins, as there were no significant differences in the competitive indices observed (Figure 5). Therefore, α-defensins do not appear to be significantly involved in selection against Ara4N-deficient bacteria.

### The Early Immune Response to *S.* Typhimurium Differs Depending on the LPS Modifications Present

Since the virulence defect of Ara4N-deficient *S*. Typhimurium does not appear to involve intestinal pH, invasion, CRAMP, or intestinal α-defensins, the early immune response initiated within the intestine may be responsible. To investigate the early immune response to WT and Ara4N-deficient *S.* Typhimurium, BALB/c mice were infected orally with 0.1 ml PBS containing 10^8^ CFU of WT (JSG210), Δ*pmrA,* or Δ*pmrF S.* Typhimurium. As a negative control, some mice were mock infected with 0.1 ml PBS. Mice were euthanized at 12, 24, and 48 hours post infection, and Peyer’s patch tissues were collected for qRT-PCR analysis of mouse immune-related gene expression, and immunohistochemical staining for infiltrating neutrophils and macrophages.

**Figure 6:**
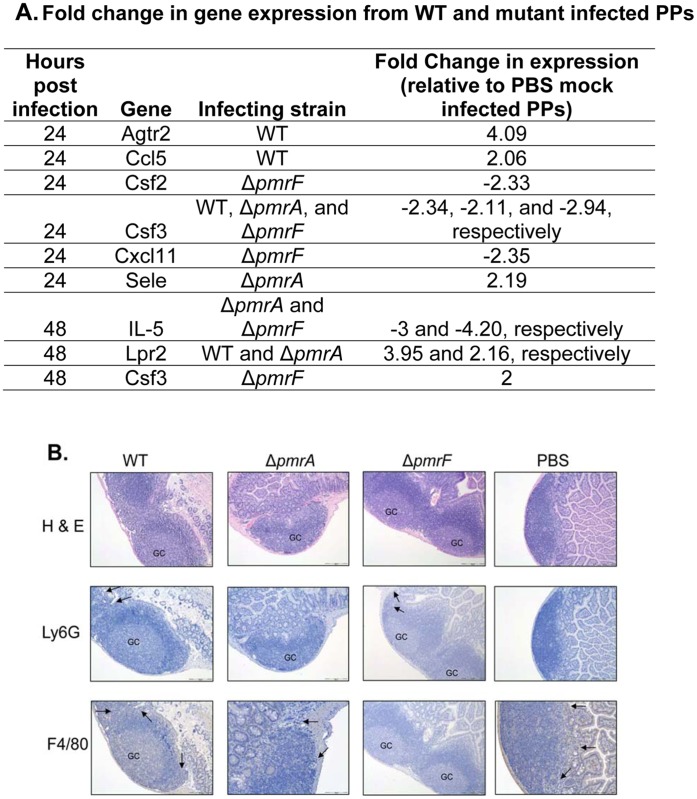
Immune response to oral infection with 10^8^ CFU of WT, Δ*pmrA,* or Δ*pmrF S.* Typhimurium. (A.) Significant changes (2-fold or greater relative to uninfected controls) in immune-related gene expressionfrom mouse Peyer’s patches infected with WT, Δ*pmrA,* or Δ*pmrF S.* Typhimurium relative to PBS mock-infected Peyer’s patches. (B.) Hematoxylin and Eosin staining, and immunohistochemical stainging of Peyer’s patches infected with WT, *pmrA, pmrF S.* Typhimurium, or mock-infected with PBS for 48 hrs. Neutrophils were stained using Ly6G antibodies. Macrophages were stained using F4/80 antibodies. GC- germinal center.

Infection with WT or mutant strains of *S.* Typhimurium did not induce significant infiltration of neutrophils or macrophages at any time point (Figure 6). Overall, Peyer’s patches infected with WT, Δ*pmrA,* or Δ*pmrF*, or mock-infected with PBS showed little to no changes in histological appearence, with the exception that the formation of germinal centers was noted in infected, but not mock-infected Peyer’s patches (Figure 6). Infection with WT or mutant strains of *S.* Typhimurium also did not induce significant changes in gene expression at 12 hrs post infection (data not shown). By 24 and 48 hrs of infection, 2-fold or greater changes in gene expression were observed in infected Peyer’s patches (Figure 6A). Infection with WT or mutant *S.* Typhimurium appeared to induce modest dysregulation of immune-related gene expression. The most prominent host gene alterations involved IL-13, IL-5, Agtr2, and Lpr2 (Figure 6A). Recent publications have identified a novel immune cell population called natural helper cells, which are found in fat-associated lymphoid clusters present in the mesenteric tissues surrounding the intestines. These natural helper cells were found to be the predominant source of the T_H_2 cytokines, IL-5 and IL-13 [20]. As the genes for both of these cytokines were initially identified from our qRT-PCR data, IL-13 KO mice were obtained from collaborators.Unfortunately, upon directed repeats of this qRT– PCR data with IL-13 and IL-5 specific primers, the IL-5 array results were confirmed, but the IL-13 results were not. Re-examination of the original IL-13 data showed a flaw in the statistical analysis, leading to the false impression of IL-13 dysregulation. Although our qRT-PCR data did not support further investigation into the role of IL-13 in the attenuation of Ara4N-deficient *S.* Typhimurium, we proceeded with survival studies, expecting no role for IL-13.

**Figure 7:**
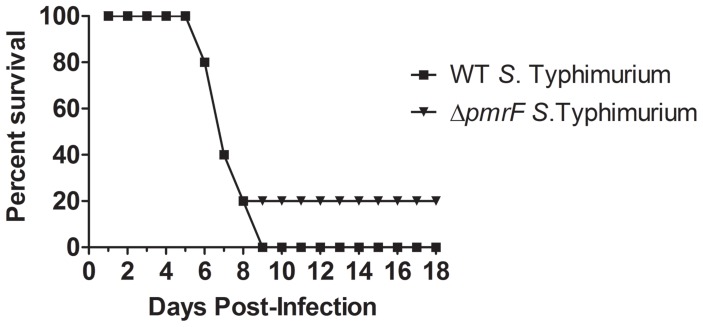
Survival of IL-13 KO BALB/c mice infected with WT or Δ*pmrF S.* Typhimurium. IL-13 KO mice on a BALB/c background were infected by oral route with 10^4^ CFU of WT or Δ*pmrF S.* Typhimurium (n = 5 mice), and were monitored for morbidity and mortality.

**Figure 8:**
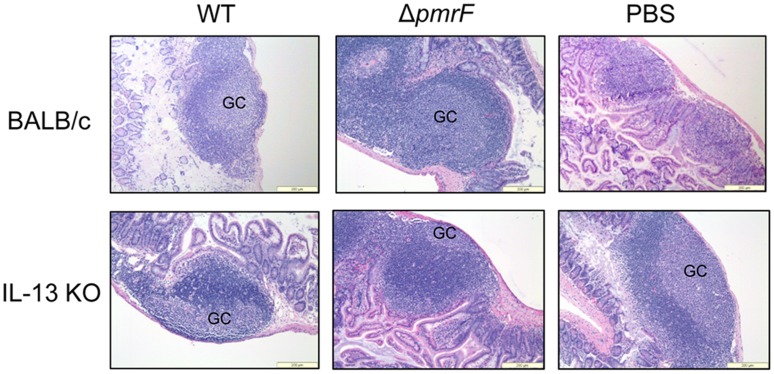
Histology of BALB/c and IL-13 KO Peyer’s patches infected with WT or Δ*pmrF S.* Typhimurium. Hematoxylin and Eosin staining of Peyer’s patches infected orally with 10^8^ CFU of WT or Δ*pmrF S.* Typhimurium, or mock-infected with PBS for 48 hrs. GC- germinal center.

Surprisingly, oral infection of IL-13 KO mice with Ara4N-deficient *S.* Typhimurium demonstrated near-WT levels of mouse mortality (Figure 7), which was dramatically more than the control BALB/c mice infected with the Ara4N-deficient strain (Figure 2). This finding suggests that although IL-13 did not appear to be transcriptionally dysregulated in the early stages of infection, it plays a role in selection against the Δ*pmrF* strain in vivo. Histological analysis revealed that Peyer’s patch tissues from WT and Δ*pmrF*-infected IL-13 KO mice looked similar to Peyer’s patch tissues collected from WT and Δ*pmrF*-infected BALB/c mice (Figure 8). The PBS mock-infected Peyer’s patches from IL-13 KO mice differed from WT BALB/c mice, as the formation of germinal centers was noticed in the PBS mock-infected Peyer’s patches from IL-13 KO mice, but not the BALB/c mice (Figure 8). However, the Peyer’s patch and surrounding intestinal tissues of infected IL-13 KO and BALB/c mice appear similar in overall tissue architecture, thus not providing a rationale for the near-WT levels of virulence of the Ara4N-deficient mutant during oral infection. Additional studies are needed to understand the role of IL-13 during *Salmonella* infection, and the mechanisms by which Ara4N-deficient *S.* Typhimurium is attenuated by oral, but not intraperitoneal infection.

## Discussion

Bacteria have evolved many mechanisms to avoid detection and killing by CAMPs and other effectors of the host immune system. Such mechanisms include LPS modification. Tight control of these bacterial resistance mechanisms through TCRSs allows *Salmonella* to respond quickly and efficiently to constant changes in local stressors and host immune factors, including CAMPs.

In this work, in vivo assays were performed to examine the temporal and spatial location of host defenses (CAMPs and immune responses) that might be responsible for inhibiting oral virulence of a CAMP-sensitive mutant *S.* Typhimurium and to analyze the bacterial response to specific host factors in the intestine. Susceptible WT (FvB, BALB/c and C57BL/6) mice and mutant mice with altered intestinal innate immunity, specifically CRAMP KO and MMP7 KO mice, were infected with *S*. Typhimurium to determine the potential role of these CAMPs during infection. Further experiements involving BALB/c or IL-13 KO mice were performed to investigate the intestinal immune response to WT and LPS modification deficient *S.* Typhimurium.

Gunn et al. [12] provided the basis for the experiments described here with the observation that an Ara4N-deficient *S*. Typhimurium strain (Δ*pmrF*) exhibits a virulence defect upon oral, but not IP, infection in susceptible mice. This mutant also has decreased resistance to polymyxin B [12]. We confirmed and extended these findings in this work, additionally showing that a PmrA^c^ strain highly modified with Ara4N is as virulent as the WT strain. These findings suggested that *Salmonella* LPS modification mutants may be targeted by host defenses (e.g. antimicrobial peptides) early during infection, before the bacteria are engulfed by macrophages [12].

Several possible explanations for the decreased oral virulence of Δ*pmrF S.* Typhimurium were investigated, including highly acidic conditions like those found in the stomach and potential invasion defects. Δ*pmrF S.* Typhimurium did not experience a survival defect compared to WT *S.* Typhimurium when grown in pH-buffered media in vitro [12]. The Δ*pmrF* and Δ*pmrA* mutants both exhibited WT levels of *Salmonella* pathogenicity island I secreted effectors and Δ*pmrF* exhibited a near-WT rate of invasion of T84 cells. Based on these results, CAMPs were tested as potential in vivo factors that participate in elimination of this LPS modification mutant in the intestine.

The addition of Ara4N to LPS may also confer a survival advantage by helping to mask the negative charge of the bacterial outer membrane, allowing bacteria to avoid recognition by host intestinal immune defenses such as positively-charged CAMPs. Recent work has demonstrated that modification of the phosphate groups present in the lipid A portion of the LPS molecule can dramatically alter the potency of LPS [21]. It is also possible that the addition of Ara4N to the phosphate groups present on the lipid A portion of LPS alters the ability of Toll-like receptor 4 (TLR4) to recognize and respond to LPS, and ultimately interferes with the ability of the immune sytem to clear *Salmonella* infection.

The work described here expands on previous CAMP studies, many of which have been performed in vitro with the findings used to speculate about the function of these molecules in vivo. Relatively few studies have confirmed whether or not these assumptions are accurate in vivo. Among those that have performed these studies, Rosenberger et al. examined the interaction of murine CRAMP with *Salmonella* and found that CRAMP expression was induced upon *S*. Typhimurium infection of macrophages [22]. CRAMP was found to inhibit *Salmonella* growth both in vitro and in vivo. In addition, Δ*phoP S.* Typhimurium survived better in CRAMP-deficient vs. WT macrophages [22]. Salzman et al. provided further evidence that CAMPs are involved in host protection against *Salmonella* by creating a transgenic mouse model that expresses human α-defensin 5 (HD-5) from murine intestinal crypts [23]. These transgenic mice demonstrated enhanced survival upon oral infection with WT *S*. Typhimurium compared to mice that did not express HD-5 [23]. However, in this work, we show that in competition assays in WT, CRAMP KO and MMP7 KO mice, CRAMP and α-defensins (cryptdins) individually do not appear to play a major role in elimination of Ara4N-deficient *Salmonella* in the examined tissues/organs in vivo.

Based on the unexpected increased severity of the competition defect for the Δ*pmrF* strain in CRAMP KO mice, the immunomodulatory properties of these CAMPs may be even more important than the direct antimicrobial effects to promote clearance of pathogens such as *S*. Typhimurium [24]. These altered protective mechanisms may eliminate Δ*pmrF* more effectively in the CAMP-deficient mice due to inappropriate signaling and/or compensation for the normal role of these molecules in host defense. This host compensation also could mask any direct role of CRAMP or α-defensins in elimination of *S*. Typhimurium in vivo. Despite these caveats, the chosen mouse models represent some of the most relevant methods currently available to examine the role of these specific CAMPs in vivo. In addition to their antimicrobial and immunomodulatory effects, CAMPs have also been shown to be an environmental signal activating the PhoP and PmrA regulons [25]. We recently demonstrated that CRAMP activates these regulons in the mouse intestine, further suggesting an important signaling role for CAMPs in vivo [10].

Or the bacterial side of the host-pathogen interaction, in addition to increased CAMP resistance, LPS modification also helps *Salmonella* evade other aspects of the host innate immune response. The PhoP-regulated genes *pagP* and *pagL* encode proteins involved in addition of palmitate and 3-*O*-deacylation of the lipid A, respectively. These modifications inhibit lipid A-induced TLR4 activation, allowing *Salmonella* to avoid detection by the host immune system [26], [27], [28]. PrmA- and PhoP-mediated LPS modifications have also been shown to dramatically affect both the innate and adaptive immune responses in *Salmonella* as well as other Gram negative pathogens [29], [30], [31]. Thus, the absence of LPS modifications in the Δ*pmrF* strain may also alter the immune response in a way that selects against this strain.

Further investigation into the early intestinal immune response to orally administered *S.* Typhimurium suggest that WT and LPS modification mutant strains induce only limited changes in the expression of immune-related genes such as IL-5 and Lrp2, but do not induce significant changes in innate immune cell migration into intestinal tissues. These findings indicate that although the changes in gene expression are not extreme, strains that differ in LPS modification can trigger an altered immune response. Preliminary investigation into the role of IL-13 found that the presence of this cytokine can influence the outcome of infection. The Ara4N-deficient *S.* Typhimurium was nearly as virulent as WT *S.* Typhimurium during infection of IL-13 KO mice, but was significantly attenuated in BALB/c mice. Although further investigation is needed to determine the role of IL-13 during *Salmonella* infection, it is possible that the absence of this cytokine lowers mucus production, or alters the development of a productive immune response, leading to survival of the otherwise attenuated Ara4N-deficient strain [20], [32]. Host pathogen interactions are very complex, and it is likely that additional factors are involved in the attenuation of Ara4N-deficient *Salmonella.*

The results of the experiments presented here indicate that CAMPs have a limited direct role in elimination of *S*. Typhimurium in vivo, and highlights the importance of immune factors such as IL-13 and other uncharacterized innate immune components in the ability of the host to mount an effective immune response to invading *Salmonella*. Multiple CAMPs and other host immune defenses (both known and unknown) likely have an additive or synergistic effect against both mutant and WT *Salmonella* that exceeds the actions of any one individual CAMP in vivo. Continued investigation into *S.* Typhimurium TCRSs, virulence gene regulation and LPS modification in vivo will help determine how bacterial pathogens evade killing and thrive in hostile host environments, which may lead to the production of novel vaccines or CAMP-based preventative therapies.
